# Understanding the Quality of Life and Its Related Factors in Orthodontics Postgraduate Students: A Mixed Methods Approach

**DOI:** 10.3390/dj11020039

**Published:** 2023-02-06

**Authors:** Laura V. López-Trujillo, Sara C. López-Valencia, Andrés A. Agudelo-Suárez

**Affiliations:** 1 Orthodontics Program, Faculty of Dentistry, Vision de las Americas University Foundation, Medellin 050010, Colombia; 2 Faculty of Dentistry, University of Antioquia, Medellin 050010, Colombia

**Keywords:** quality of life, mental health, psychological well-being, dental students, orthodontics, research technics, multivariate analysis, qualitative research

## Abstract

This study analyzed the academic, sociodemographic, and labor conditions related to the quality of life (QOL) of orthodontics postgraduate students in Colombia. A mixed study (explanatory sequential design) was conducted. An online cross-sectional survey (*n* = 84; 64.3% females) was carried out with sociodemographic, academic, social support, health, labor, and QOL (WHOQOL-BREF) variables. Descriptive, bivariate analyses, and multivariate linear regression were performed. Focus groups (FGs) delved into aspects of relevance regarding QOL and determinants, through qualitative content analysis and triangulation of information. The median score in the four WHOQOL-BREF dimensions surpasses 50 points, with the highest score being in the psychological dimension (62.5 ± 16.7). According to the multivariate linear regression models, the variables significantly associated with QOL scores were playing sports, being married/living together, normal BMI, low social support, and medium/low socioeconomic status. The qualitative results explained the determinants of QOL in the personal, academic, and social dimensions of the participants. The discourses showed that the postgraduate course represents a resignification of their life, where their QOL is affected by the difficulties of their academic development, by the difficulty of reconciling the personal academic load with their affective, work, and social life, and by the stress they experience in their staff process. In conclusion, the participants’ QOL was moderate and affected by different factors. The findings highlighted the importance of mental health promotion and well-being strategies in students of orthodontic postgraduate training programs in Colombia for improving QOL.

## 1. Introduction

Quality of life (QOL) as an individual and societal concept is not a new concept [1]. Different studies in medical and social areas have been evidenced by assessing the quality of life perception of different groups in terms of their appraisement (good or bad) in various dimensions and scales [2,3]. This concept depends on subjective elements from an individual’s experience and recognizes the influence of collective and cultural factors as well as the labor, economic, and political context [1,2,3].

Health personnel could be exposed to personal, social, and labor situations that impact their QOL [4,5,6]. In addition, the situation experienced in the last two years due to the COVID-19 pandemic has altered the world order and has especially impacted the physical and mental health of these workers [7]. The dental profession is not an exception and, in fact, some studies evaluate the QOL in general dentists and specialists who work in public and private sectors [8,9,10]. Some of this research has considered the proposal guidelines by the World Health Organization Quality of Life assessment group (WHOQOL) and employed instruments that would be applicable cross-culturally. In addition, it primarily focused on four domains: physical, psychological, social, and environmental [11]. Other areas of research on this topic included well-being at work [12], or the relationship between QOL and physical, mental, and psychosocial health status.

Postgraduate students in clinical dental areas are presented with high demands and academic loads, and the QOL could be affected by personal and social situations and thus impact their physical and mental health status. A simple literature search on PubMed about the topic can reveal that there are some factors to study in this population. For instance, in dental students, the presence of anxiety and sleep disorders are considered as factors related to the QOL [13]. Similarly, other research mentions other mental health, wellness, and social support indicators [14]. In addition, the QOL is studied in bachelor/undergraduate students by using a cross-sectional survey [15]. Despite the presence of some research focused on assessing QOL, more studies are needed that delve into the factors of the family, academic and social contexts, which are specific for students who receive postgraduate training. Research in mental aspects and QOL in this kind of postgraduate students is scarce when compared with research in undergraduate dental students and dental professionals [8,9,10,15].

The study of complex phenomena through modern methodologies that capture social reality from different perspectives is an important challenge for dentistry. In this sense, both qualitative research [16] and mixed methods approaches [17] recognize the perceptions of health and QOL of those directly involved. A 2018 systematic review to map the literature using a blended approach to oral health uncovered nine articles on oral health outcomes. The authors concluded about the benefits of using this methodology for addressing the population’s oral health outcomes and highlighted the importance of conducting more studies on this topic [18]. In this particular case, understanding and identifying the factors that influence the QOL in the unique realities of dental students will allow the establishment of strategies to promote mental health and well-being and diminish burnout in educational institutions [19,20].

Accordingly, this study aims to analyze the academic, sociodemographic, and labor conditions related to the QOL of orthodontic postgraduate students in Colombia.

## 2. Materials and Methods

### 2.1. Design

A sequential explanatory design (mixed-methods study) was conducted [17]. This methodology has been applied in previous research [6]. This design allows qualitative tools that could explain what was observed in the first quantitative phase. Fieldwork was carried out between June 2021 and April 2022. All the components of this study are referenced below.

### 2.2. Quantitative Sub-Study (Cross-Sectional)

An online survey was applied for orthodontic students in Colombia. No-probabilistic sampling methods were carried out, and participants who freely and voluntarily answered the survey were included. For that purpose, diverse strategies were applied such as emails provided by universities, use of social networks (Facebook and WhatsApp) and using snowballing sampling methods through referrals and forwarding of the questionnaire to other students. The final sample was 84 (54 females, 64.3%). The questionnaire was self-administered by using the Google Forms tool (available upon request). All surveys were anonymous and confidential. A pilot study was conducted with a sample of 10 dentists to improve interpretability and evaluate the completion time and internal consistency.

The primary outcome was quality of life (QOL), as measured by the WHOQOL-BREF [11]. This is a generic and validated questionnaire created by the WHO Quality of Life Study Group and made up of 26 items divided into four main areas: physical health, psychological health, social relationships, and environment. We used the main recommendation of the study group for the analysis of these domains and their scores. Explanatory variables were included: sociodemographics, academic, labor conditions, self-perceived general health, and mental health (measured with the 12-item version of the General Health Questionnaire GHQ-12) [21]. Social support was evaluated by using the Duke-UNC-11 questionnaire. This instrument, which consists of 11 elements, evaluates the perceived functional or qualitative social support. Each element is scored according to a frequency rating of 1: “Much less than I would like” to 5: “As much as I would like”. The score was calculated by adding the responses to each item together, a higher score indicating greater social support. The threshold for low levels of social support is the 15th percentile, resulting in a score of 32 or less [22]. 

Cronbach alpha coefficients were calculated using the WHOQOL-BREF, GHQ-12 and Duke-UNC-11 instruments of the sample to assess their reliability. The following values were obtained: (1) WHOQOL-BREF: 0.717; (2) GHQ-12: 0.855; and (3) Duke-UNC-11: 0.916 (When the values are close to 1.00, they indicate higher reliability). 

All variables underwent descriptive analysis. The normality distribution of the quantitative variables was evaluated (Kolmogorov–Smirnov test). Bivariate analysis has been performed for QOL domain scores with explanatory variables according to their nature (Mann–Whitney U test for dichotomous variables, Kruskal–Wallis test for polychotomous variables, and the Spearman correlation for quantitative variables). A linear multivariate regression analysis was carried out in order to evaluate the association of the explanatory variables on each of the dimensions of WHOQOL-BREF and to identify possible predictors of their scores. Belonging was determined by evaluating the compliance with the assumptions of linearity, non-collinearity and normality, constant variance, and correlation of residuals. All of the analyses used a level of statistical significance of <0.05. SPSS software version 22.0-IBM^®^ was used to carry out all of the analyses.

### 2.3. Qualitative Sub-Study (Focused Ethnographic Perspective)

A qualitative approach (under the focused ethnographic perspective) was conducted using three focus groups (FGs) and 17 orthodontics students that previously completed the survey participated (selected for convenience). The research team produced a guide for use in the FGs that indicated a series of topics related to the QOL and its determinants. Similarly, some relevant quantitative findings are deepened through the opinion of participants, and the information that was confusing or contradictory was expanded. The FGs were conducted by one member of the research team (A.A.A-S.), who is a Ph.D. in public health and has expertise in qualitative methods. The FGs lasted between 60 and 90 min and were digitally recorded and transcribed verbatim. Concerning the situation of the COVID-19 pandemic, and to obtain the participation of periodontists from various parts of Colombia, remote means (Microsoft Teams) were used.

Analysis of the narrative content led to the identification of important text elements and information trends found in the participants’ speeches. Analyses of the data were conducted by all authors and were reviewed and compared. Atlas.Ti 8.0 software was used for analyzing the transcribed data. The text fragments were tagged under 126 codes and then grouped into 3 categories.

### 2.4. The Methods Integration Approach

The integration of the two sub-studies were integrated using triangulation [17,23]. Two conceptual maps and a word cloud were formulated, identifying the factors related with the academic experience and those influencing QOL according to individuals’ opinions and social factors related to the particularities of this students’ group in Colombia.

### 2.5. Ethics

Individuals gave their consent, which was explained on the first page of the survey and was read and approved for the FGs. Confidentiality was ensured throughout the research process, in compliance with standard regulations. The study protocol was approved by the Bioethics Research Committee of the Faculty of Dentistry of the University of Antioquia (Act 03/2021, Concept Nº 75).

## 3. Results

### 3.1. Quantitative Findings

#### 3.1.1. General Profile of Participants

Table S1 (Supplementary Materials) shows the sociodemographic, academic, occupational, health, and QOL characteristics of the target population. The median age was 29 ± 4 years. Almost two-thirds were women. Slightly more than 70% of the participants were single, and slightly more than one-third were from middle or low socioeconomic statuses. Almost 60% lived in their own homes; slightly more than half owned a vehicle; and almost two-thirds came from nuclear families (father, mother, and siblings). Regarding labor conditions, they have a median of 6 ± 5 years of professional experience; almost 60% are currently working and have several workplaces. Considering academic variables, their daily schedule in educational activities is 7 ± 3 h per day, with a median of 40 ± 35 h per week; three-quarters spend on average up to COP 3 million (approx. USD 750) per month in their postgraduate studies. Overall, 92% consider that there is an imbalance between their academic and free time; a little more than half are proficient in a foreign language; and most are satisfied with the postgraduate experience, although they consider it stressful.

Regarding QOL according to the WHOQOL-BREF instrument, the median score in the four dimensions surpasses 50 points, with the highest score being in the psychological dimension (62.5 ± 16.7). Finally, regarding health and social support indicators, only a third indicated that they practice sports, a quarter reported overweight/obesity, a third perceived their general health as poor, a little more than half referred to their mental health as poor, and a little more than 10% perceived low social support (Table S1).

#### 3.1.2. Relationships between QOL and Sociodemographic, Academic, Occupational, and Health Variables

Table 1 shows the different bivariate correlations between the QOL dimensions of the WHOQOL-BREF instrument and the quantitative variables included in the study. There were no statistically significant correlations in the sample of participants.

Table 2 shows the bivariate comparison between the QOL domains of the WHOQOL-BREF instrument and the qualitative variables included in the study. In the case of the physical dimension, statistically significant differences were found in the median scores for the variables: owning a vehicle (>yes), practicing sports (>yes), self-perceived health (<poor), mental health (<poor), and social support (<low). Concerning the psychological dimension, statistically significant differences were found with the variables practicing sports (>yes), and social support (<low). When analyzing the social relationships dimension, statistically significant differences were found with the family type (<living alone), study–leisure time balance (<unbalanced), self-perceived health (<poor), and social support (<low). Finally, according to the environment dimension, statistically significant differences were found for the variables socioeconomic status (>high), owning a vehicle (>yes), type of family (<living alone), study–leisure balance (<unbalanced), foreign language proficiency (>yes), postgraduate satisfaction (>satisfied), the stress level in postgraduate school (<stressful), practicing sports (>yes), self-perceived health (<poor), mental health (<poor), and social support (<low).

#### 3.1.3. Potential Explanatory Factors for QOL Dimensions

A multivariate analysis by linear regression showcased the potential factors explaining QOL that were significantly associated. For the physical dimension, social support is negatively related to QOL (having low social support decreases the score). In the psychological dimension, playing sports, marital status (being married), and having a normal body mass index were positively associated (increase QOL scores), whereas social support was negatively associated. As for the social relationships dimension, playing sports was positively associated, while living alone was negatively associated. Finally, on analyzing the environmental dimension, it was found that playing sports and marital status (being married) were positively associated with QOL, while having low social support, a medium or low socioeconomic stratum, and living alone were negatively associated (Table 3).

### 3.2. Qualitative Findings

The analysis of the focus groups (FGs) deepened the aspects observed in the quantitative results. From the participants’ perspectives, it was possible to establish two main categories, which are discussed below:

#### 3.2.1. Quality of Life: Definitions, Determinants, and Satisfiers

*“So those specific life conditions and those specific commitments from the, let’s say, personal point of view are the most important factor that makes the difference between the quality-of-life perception of different residents, considering that we all have something in common at this moment, which is the residency or the postgraduate degree, but there are certain things in each one’s life that can create those differences in regard to the perception of quality”.*

(FG 3)

**Figure 1:**
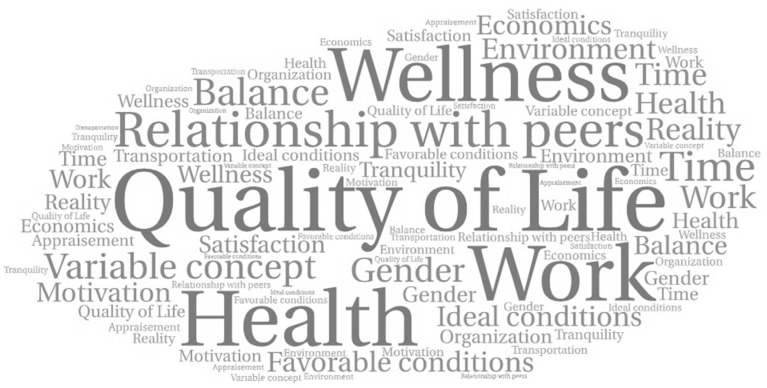
Word cloud for the concept of QOL according to the participants’ discourses. Elaborated by using wordart.com (free online tool: accessed on 5 October 2022).

Quality of life denotes a multidimensional and dynamic concept. The participants’ discourses made it possible to establish in common 20 keywords that define QOL (Figure 1) as well as establish, in general, flourished words such as well-being, balance, satisfaction, and personal experience, among others. It is recognized that this is a dynamic and variable concept of which there are standards and scales of values that allow it to be recognized and evaluated subjectively (Supplementary Materials: Table S2, 1a).

**Figure 2:**
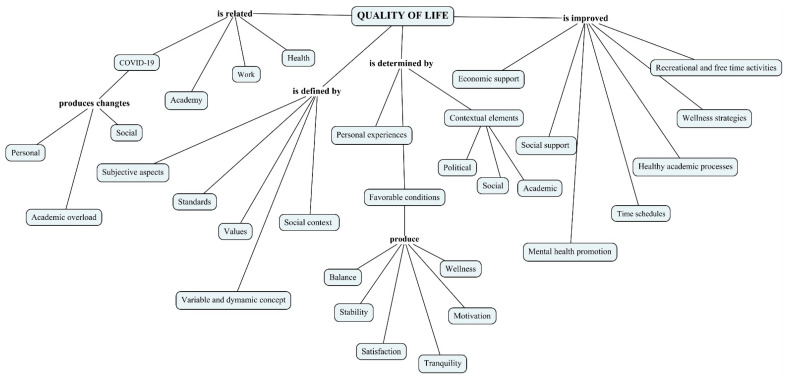
Conceptual map for “Quality of life: definitions, determinants, and satisfiers”. Elaborated by using Cmaptools 6.03.01.

Figure 2 shows the map of relationships established with the concept of QOL, understanding that it is related to elements of personal experience, but other situations emerge that are related to the social, political, economic, and academic contexts (Table S2, 1b,c). As satisfiers, the participants highlighted elements such as the role of social and family support networks, having spaces for recreation and free time, strategies for mental health promotion, and “healthy” academic processes that allow for greater interaction between personal and academic aspects (Table S2, 1d–f). The COVID-19 pandemic is mentioned as a conditioning factor in QOL, as there was a range of changes in the dynamics of teaching towards virtual strategies, causing a greater academic load, for which many postgraduate residents were not prepared. To this is added all the factors related to uncertainty in the face of an unknown disease, which caused changes in the social dynamics and the biosecurity protocols in the clinic, among other situations (Table S2, 1g,h).

#### 3.2.2. A Roller Coaster Ride: The Postgraduate Training as a Personal and Academic Life Project

*“I want to say something that has to do with the academic part obviously and it’s the patients, because I don’t know if in your universities it is like this, but in mine we have to get the patients, so you have to constantly be after people, looking for someone to be your patient and in the university, supposedly people goes there, but that’s a lie, no one goes there and it doesn’t matter to them if you got them, if you paid to them, if you didn’t, but is very difficult”.*

(FG 2)

**Figure 3:**
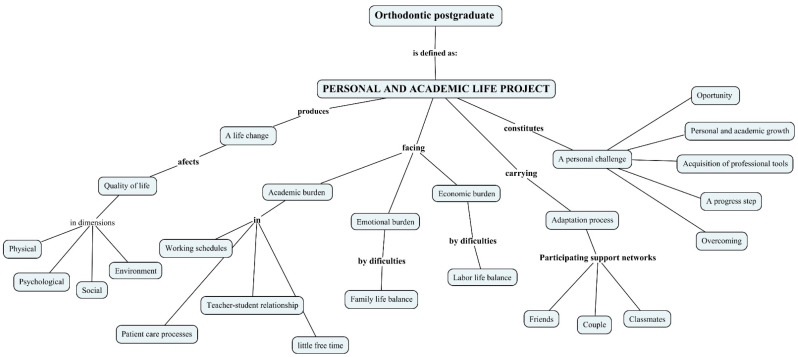
Conceptual map for “a roller coaster ride: the postgraduate training as a personal and academic life project”. Elaborated by using Cmaptools 6.03.01.

From the participants’ experience, the postgraduate course in orthodontics is a personal, academic, and professional project, as shown in Figure 3. It produces a change of life-affecting QOL in its different dimensions; in the words of the participants, “one changes family”. The students face academic, personal, and economic burdens since it implies changes in schedules, leaving work to concentrate on their studies, and problems in reconciling their family, emotional, and social lives (Supplementary Materials: Table S2, 2a). Additionally, they mentioned factors inherent to the academic process in universities and highlighted an important element that has to do with the teacher–student relationship, which is fundamental to QOL and the mental health of the students (Table S2, 2b–d).

However, despite the difficulties, the participants considered that an adaptation process can be carried out in which support networks play an important role and that studying for a postgraduate degree is an opportunity for personal and professional growth and improvement, helping them advance one step further in their professional training in comparison to general dentists (Table S2, 2e,f).

## 4. Discussion

### 4.1. Main Findings

The participants reported QOL scores in different dimensions that range between 50 points for the physical dimension and 63 points for the psychological dimension. However, the bivariate and multivariate analyses show that the QOL is associated with different academic, sociodemographic, occupational, and health factors that exert a positive or negative influence. Although the QOL is perceived as moderate, the participants’ discourses in the FGs showed that the postgraduate course is a personal and academic project where a deterioration of mental health and QOL is evidenced, where subjective and those related to the educational context intervene, and other factors such as difficulties in reconciling family and social life with a higher academic and scientific workload also impacted on the decreasing of QOL. This situation makes the postgraduate experience, on the one hand, rewarding for achieving a step in their professional life, but on the other hand, a process of constant change and adaptation that causes frustration in certain specific aspects.

### 4.2. Possible Explanations of the Findings: What the Scientific Literature Tells Us on This Topic

The scores given in the different domains in the QOL by orthodontic postgraduate students show a close relationship with their particular experiences and the groups in which they are inserted and have social cohesion. Thus, when these results were compared with other contexts, certain differences are made evident. For example, the QOL scores in our study were much lower when compared to a study conducted with orthodontists who had already graduated from the city of Medellin (Colombia) [6]. The same situation was observed when compared with other studies, which included general dentists and specialists, carried out in the United Arab Emirates [8] and India [9]. A study of dental students in the United States showed scores between 63 and 70 (by using means) [15]. Similarly, other research conducted in Singapore evaluated the WHOQOL-BREF and conducted a cross-cultural comparison with other studies on dental students, and the findings were quite comparable to our study [24]. Lastly, we draw attention to the findings observed in postgraduate medical students working in private and public hospitals in Pakistan [25], where the scores were higher when compared to the Colombian study in orthodontics students. The results can be interpreted according to the academic and personal processes experienced by the study participants and that were related to the characteristics of the postgraduate training style in Colombia.

The QOL from the perception in the speeches of the participants is defined as multifactorial and multi-dimensional. Studying orthodontics becomes an academic and personal process where specific factors come together that affect QOL positively or negatively. For instance, a qualitative study conducted in Germany [26] found through dialogue in FGs that the high demands associated with medical studies come into conflict with academic studies and other demands related to private life. In addition, a lack of resources for recovery and certain personal characteristics could contribute to the generation of stress among medical students, reducing their well-being. This situation would undoubtedly decrease QOL. Another study carried out with master’s students revealed some representations of the subjective aspects of QOL, such as its meaning and concept, and its particular expressions such as intimacy, social and family time, emotional expression and perceived health, mood, perceived security, self-esteem, productivity, and fulfillment of expectation [27]. In this sense, the QOL of orthodontic students configures new dynamics in different scenarios of their environment, and this aspect regarding the determinants of QOL at different levels is reflected in the participants’ speeches.

Social support networks (friends, family, colleagues) are essential QOL predictors in the study population. In multivariate models, the perception of low social support and living alone are associated with lower QOL scores, while being married/cohabitated is associated with higher QOL scores. This situation was corroborated by the qualitative findings in the FGs, where the participants’ discourses highlighted the support of friends and family as satisfying elements of QOL. In this regard, a study conducted on Chilean university students shows how social support plays a protective role for symptoms related to stress and anxiety [28], and a similar situation was observed in dental students from Canada [29]. A study carried out on dental students in Turkey showed that high social support is positively related to a greater sense of coherence [30], an element that, from Antonovsky’s salutogenic theory, is constituted as a promoter of health and well-being. This factor is considered an indirect indicator of QOL.

Quantitative analyzes carried out in the students’ sample and the discourses provided in the FGs show a close relationship between the QOL and health indicators. In fact, one third of the participants reported poor self-perceived health, more than half reported poor mental health, and the majority reported the postgraduate studies as stressful (we found a statistically significant association with some scores of QOL as provided for the instrument). Multivariate models show a normal BMI and practicing sports as positive predictors for QOL. A Chinese study conducted on medical and dental students found that physical fitness and lifestyle are significant contributors to academic performance [31]. However, in the analysis of QOL–health, it is important to consider the role of factors that can contribute to improving both conditions in postgraduate students. For example, there are studies focused on the factors that favor resilience (the ability to bounce back or recover from major life stressors) [32]. Discourses in the FGs referred to some alternatives that produce wellness and produce QOL, as provided by institutional strategies for postgraduate students. Improving QOL could be accomplished by other conceptual frameworks such as positive mental health that uses the basis of health promotion [33].

Qualitative findings show that QOL could be affected by the COVID-19 pandemic. Specifically, in the academic field, an adaptation process was presented in the teaching–learning forms and the patient care protocols in this training process. This is an emerging fact since the survey did not contemplate specific variables that measured the impact of the pandemic, and this was not a specific objective of the study. In this regard, research conducted with dental students in Turkey shows how online teaching mechanisms taught during the pandemic impacted increased stress, anxiety, and the presence of factors that negatively affected the QOL [34]. In a cross-sectional Norwegian study aimed at mapping dental students’ experience of the study situation throughout the pandemic [35], the main results show that the pandemic affected collective and academic life, and evidence the presence of learning stressors and an increased burden. Finally, postgraduate students could have experienced stress during the mandatory social isolation, as shown by one multi-institutional study conducted in dental students from six countries: the US, Spain, Ireland, Chile, India, and Brazil [36].

### 4.3. Scope of This Study

In the interpretation of the findings, is important to highlight the importance of discussing the main strengths and limitations of this study. We recognize that, to the best of our knowledge, this is the first study focused on a particular group of orthodontic students in Colombia. The mixed methods approach allowed us to understand the factors affecting the QOL in the target population, transcending the classic biomedical point of view of seeing health–disease processes and allowing the social construction of the research problem. In addition, the data collection instruments were carefully planned and have internal validity processes, both by a pilot test and by the consistency and validation of the QOL questionnaire, as well as with the reliability and rigor in the FGs used. Nevertheless, the nature of the study design does not allow for the establishment of causal relationships, but rather associations between variables in the cross-sectional sub-study and understanding of relationships between categories of analysis in the qualitative sub-study. This type of non-probabilistic sample does not permit the results to be inferred from the general population of orthodontic students. Lastly, it seems important to mention that the findings could be affected by the time of the survey and the semiannual/annual calendar of academic activities in the different institutions where the students are enrolled.

Accepting the above limitations, this study is constituted as an input to know the social, health, and QOL aspects in specific scenarios. Complementary studies are required in Colombia, which, through more representative samples and involving other questionnaires and social, physical, and mental health indicators, allow us to understand the reality experienced by students of clinical specialties in dentistry. Further research should delve into aspects related to the curricula in the training of students of clinical specialties in dentistry from the perspective of other authors (professors, administrative staff, and patients).

## 5. Conclusions

From the perspective of the students participating in the study, QOL is perceived as moderate and is conditioned by sociodemographic factors, academic aspects during their training, and their physical and mental health situations. The predictor variables for QOL were social support, socioeconomic status, marital status, type of family, BMI, and sport practice. The postgraduate course is an academic and personal project that highlights particular and general situations and that has a role in the deterioration of the QOL, but, in turn, will allow them, in the long term, to improve their economic conditions, which will have an impact on a better QOL in the near future. Some satisfiers were found that deserve to be considered to generate strategies that contribute to the general well-being of these students from holistic and integral approaches.

## Figures and Tables

**Table 1:** Bivariate correlations between the WHOQOL-BREF dimensions of QOL and the quantitative variables in the study sample (*n* = 84).

Variables	WHOQOL-BREF Dimensions (QOL)			
	Physical ^a^	Psychological ^a^	Social Relationships ^a^	Environment ^a^
Age	−0.076	−0.070	0.017	−0.093
Years of experience as a dentist	−0.013	−0.050	−0.043	−0.098
Daily hours of face-to-face academic schedule	−0.091	0.079	0.025	0.004
Weekly study hours	−0.025	0.199	0.094	0.098
Resting days per week	−0.044	−0.081	0.071	−0.091
Annual frequency of events of academic training	−0.011	0.033	0.104	−0.067

**^a^** Spearman’s rank correlation coefficient. No significant statistically correlations were found in the sample.

**Table 2:** Bivariate comparison of the WHOQOL-BREF dimensions of quality of life and the qualitative variables in the study sample (*n* = 84).

Variables	WHOQOL-BREF Dimensions (QOL)											
	Physical			Psychological			Social Relationships			Environment		
	Me	IQR	*p*-Value ^a^	Me	IQR	*p*-Value ^a^	Me	IQR	*p*-Value ^a^	Me	IQR	*p*-Value ^a^
Sociodemographics												
Sex												
Females	48.2	21.4	0.663	66.7	16.7	0.219	58.3	25.0	0.702	59.4	15.6	0.094
Males	50.0	18.8		60.4	21.9		54.2	35.4		53.2	24.2	
Marital status												
Single	46.4	21.4	0.735	62.5	20.8	0.128	58.3	25.0	0.767	57.8	18.0	0.866
Single/Cohabitated	50.0	19.6		66.7	20.8		58.3	33.3		56.3	32.8	
Separate	50.0	---		54.2	---		50.0	---		56.3	---	
Socioeconomic status												
Low-Middle	48.2	17.9	0.630	62.5	17.7	0.068	54.2	25.0	0.233	54.7	15.6	0.025
High	50.0	25.0		66.7	16.7		66.7	33.3		62.5	29.7	
Housing												
Own	50.0	21.4	0.236	62.5	20.8	0.339	66.7	33.3	0.239	59.4	18.0	0.150
Rented	46.4	10.7		66.7	18.8		50.0	37.5		53.1	26.6	
Other	46.4	16.1		58.3	14.6		50.0	29.2		53.1	23.4	
Vehicle												
Yes	53.6	62.5	0.041	62.5	20.8	0.215	58.3	25.0	0.757	59.4	25.0	0.028
No	46.4	10.7		62.5	20.8		58.3	37.5		53.1	15.6	
Type of Family												
Nuclear	50.0	18.8	0.068	66.7	20.8	0.264	62.5	33.3	0.024	59.4	21.9	0.011
Assembled	---	---		68.8	---		75.0	---		62.6	---	
Extended	42.9	---		62.5	---		58.3	---		62.5	---	
Single-parent	48.2	26.8		60.4	14.6		66.7	27.1		64.1	16.4	
Live alone	39.3	14.3		54.2	12.5		41.7	16.7		46.9	15.6	
Labor conditions												
Currently working												
No	46.4	21.4	0.529	66.7	16.7	0.274	66.7	33.3	0.62	56.3	15.6	0.507
Yes	50.0	19.6		62.5	20.8		58.3	25.0		59.4	21.9	
Having several workplaces (n = 49)												
No	48.2	24.1	0.186	62.5	25.0	0.976	54.2	31.3	0.869	59.4	24.2	0.984
Yes	50.0	12.3		62.5	16.7		58.3	25.0		59.4	20.3	
Academic conditions												
Postgraduate monthly expenses (COP)												
≤de 3.000.000 (≤USD 750)	46.4	21.4	0.449	66.7	16.7	0.196	58.3	25.0	0.992	59.4	18.8	0.139
≥de 3.000.001 (≥USD 751)	50.0	21.4		58.3	25.0		58.3	29.2		53.1	20.3	
Study-leisure balance												
Balanced	57.1	21.4	0.094	66.7	20.8	0.380	91.7	25.0	0.043	68.8	12.5	0.019
Unbalanced	46.4	19.6		62.5	18.8		58.3	25.0		56.3		
Foreign language proficiency												
No	46.4	16.1	0.238	62.5	16.7	0.089	54.2	31.3	0.506	53.1	14.8	0.013
Yes	50.0	20.5		66.7	20.8		58.3	31.3		60.9	21.1	
Satisfaction with the postgraduate experience												
Satisfied	50.0	21.4	0.099	62.5	16.7	0.345	58.3	25.0	0.527	59.4	18.0	0.027
Unsatisfied	42.9	12.5		54.2	25.0		58.3	25.0		48.4	20.3	
Posgraduate stress level												
Non-stressful	62.5	18.8	0.037	75.0	24.0	0.156	83.3	41.7	0.105	70.3	19.5	0.039
Stressful	48.4	20.5		62.5	19.8		58.3	25.0		58.3	18.0	
Health												
Sports practice												
Yes	57.1	19.6	0.006	66.7	18.8	0.009	66.7	37.5	0.064	62.5	17.2	<0.001
No	46.3	14.3		62.5	20.8		50.0	33.3		53.1	15.6	
Body Mass Index (BMI)												
Underweight	44.6	13.4	0.156	66.7	24.0	0.773	50.0	33.3	0.32	57.8	32.8	0.676
Normal	50.0	21.4		62.5	16.7		58.3	25.0		56.3	15.6	
Overweight/obesity	50.0	16.1		62.5	29.2		58.3	50.0		56.3	31.3	
Self-perceived health												
Good	50.0	17.9	0.038	66.7	16.7	0.084	62.5	31.3	0.009	59.4	20.3	0.009
Poor	42.9	13.4		60.4	24.0		50.0	41.7		51.6	18.0	
Mental health (GHQ-12)												
Good	53.6	17.9	0.005	66.7	16.7	0.121	66.7	25.0	0.152	59.4	21.9	0.009
Poor	42.9	12.5		62.5	20.8		58.3	33.3		53.1	18.8	
Social support (Duke-UNC-11)												
Normal	50.0	21.4	0.020	66.7	20.8	0.001	66.7	25.0	0.001	59.4	18.8	<0.001
Low	42.9	14.3		45.8	20.8		41.7	33.3		37.5	15.6	

**^a^** Mann–Whitney U test for dichotomous variables, and Kruskal–Wallis test for polychotomous variables. IQR: Interquartile range.

**Table 3:** Lineal regression models for the scores of the WHOQOL-BREF dimensions of QOL according to the variables included in the study sample (*n* = 84).

WHOQOL-BREF Dimensions (QOL)	Variables Included in the Lineal Regression Model	Determination Coefficient (%)		Change of R2%	*p*-Value Change of R2%	Non-Standardized Regression Coefficient	Standardized Regression Coefficient	*p*-Value	F-Value	*p*-Value (Model)	Durbin–Watson Statistic
		Unadjusted	Adjusted								
Physical	Social support (low)	12.3	10.5	12.3	0.013	−14.075	−0.351	0.013	6.62	0.013	2.006
Psychological	Sport practice (Yes)	56.1	52.1	6.7	0.013	13.71	0.483	<0.001	14.029	<0.001	1.824
	Social support (low)					−15.332	−0.331	0.002			
	Marital status (married/cohabitated)					8.996	0.290	0.006			
	BMI (Normal)					7.741	0.263	0.013			
Social Relationships	Sport practice (Yes)	24.4	21.1	9.2	0.022	14.702	0.368	0.006	7.429	0.002	1.696
	Type of family (live alone)					−18.333	−0.304	0.022			
Environment	Social support (low)	56.5	51.4	6.1	0.018	−21.760	−0.419	<0.001	11.170	<0.001	1.840
	Socioeconomic status (Middle-Low)					−10.657	−0.273	0.011			
	Sport practice (Yes)					9.693	0.305	0.005			
	Marital status (married/cohabitated)					9.287	0.267	0.012			
	Type of family (live alone)					−12.098	−0.252	0.018			

## Data Availability

Quantitative data presented in this study are available upon reasonable request from the corresponding author.

## Supplementary Materials

The following supporting information can be downloaded at: https://www.mdpi.com/article/10.3390/dj11020039/s1, Table S1: General profile of the study sample of postgraduate students (*n* = 84); Table S2: Verbatim extracts from participants’ discourses in focus groups *(n* = 3).
