# Supplementary material for: Understanding the Quality of Life and Its Related Factors in Orthodontics Postgraduate Students: A Mixed Methods Approach
Source: Dent J (Basel). 2023 Feb 6;11(2):39. doi: 10.3390/dj11020039 (PMC9955001; doi:10.3390/dj11020039)
Supplement: Supplementary file 1 [file dentistry-11-00039-s001.zip › dentistry-2112089-supplementary.pdf]

Table S1. General profile of the study sample of postgraduate students (n=84)

Variables	n	%
Sociodemographic		
Sex		
Females	54	64.3
Males	30	35.7
Age[a]		
Median (IQR)	29.0	4.0
Marital status		
Single	60	71.4
Married/Cohabitated	21	25.0
Separate	3	3.6
Socioeconomic status		
Low- Middle	56	69.0
High	30	31.0
Housing		
Own	50	59.5
Rented	25	29.8
Other	9	10.7
Vehicle		
Yes	47	56.0
No	37	44.0
Type of Family		
Nuclear	54	64.3
Assembled	2	2.4
Extended	3	3.6
Single-parent	10	11.9
Live alone	15	17.9
Labor conditions		
Years of experience as a dentist[a]		
Median (IQR)	6.0	5.0
Currently working		
No	35	41.7
Yes	49	58.3
Having several workplaces (n=49)		
No	28	57.1
Yes	21	42.9
Academic conditions		
Daily hours of face-to-face academic schedule[a]		
Median (IQR)	7.0	3.0
Weekly study hours[a]		
Median (IQR)	40.0	35.0
Resting days per week[a]		
Median (IQR)	1.0	1.0

Postgraduate monthly expenses (Colombian peso)[b]		
≤ de 3.000.000 (≤ U$ 750)	63	75.0
≥ de 3.000.001 (≥ U$ 751)	21	25.0
Study-leisure balance		
Balanced	7	8.3
Unbalanced	77	91.7
Foreign language proficiency		
No	40	47.6
Yes	44	52.4
Satisfaction with the postgraduate experience		
Satisfied	72	85.7
Unsatisfied	12	14.3
Postgraduate stress level		
Non-stressful	4	4.8
Stressful	80	95.2
Annual frequency of events of academic training[a]		
Median (IQR)	9.0	20.0
Quality of Life (QOL)		
Physical[a]		
Median (IQR)	50.0	21.4
Psychological[a]		
Median (IQR)	62.5	16.7
Social relationships[a]		
Median (IQR)	58.3	25.0
Environment[a]		
Median (IQR)	56.3	18.8
Health		
Sports practice		
Yes	29	34.5
No	55	65.5
Body Mass Index (BMI)		
Underweight	6	7.1
Normal	57	67.9
Overweight/obesity	21	25.0
Self-perceived health		
Good	56	66.7
Poor	28	33.3
Mental health (GHQ-12)		
Good	39	46.4
Poor	45	53.6
Social support (Duke-UNC-11)		
Normal	75	89.3
Low	9	10.7

[a] Kolmogorov-Smirnov test for Normality. [b] Dollar values between parenthesis (at the time of the fieldwork). IQR: Interquartile range

Table S2. Verbatim extracts from participants' discourses in focus groups (n=3)

Categories	Key words	Verbatim extracts form participants' discourses
**1) Quality of life: definitions, determinants, satisfiers**	Variable concept	*a) "Because maybe for me quality of life can be emotional stability and, I don't know, economic stability, but for someone else it can be health. It can be a very different concept for each person, depending on its economic, familiar, social situation" (FG 1)*
	Conditions for quality of life	*b) "I think that it's about having stability and having positive conditions in general: it can be in the family aspect, in the personal aspect, the emotional aspect, having stability. For people like us, doing the residency and working too, it's about having work stability, an academic stability, meaning that the academy doesn't turn into a problem, but rather into a rewarding moment. Then finally perhaps the economic part which is like the support, and it's quite important for us at this moment as professionals who are going through the residency." (FG 3)*
		c) "I think that what can vary is your environment, the pressure that you endure, basically that's what could alter your quality of life: the pressure, the stress and the environment where you are. It's focused on that" (FG 2)
	Satisfiers	*d) "Well, I do believe that in the family they can help, but also the colleagues are a very important support because they are the ones who really understand the situation you are going through" (FG 1)*
		e) "Well, the social welfare activities that the University does, you don't really have time to be able to take advantage of them, then on some occasions they are done but, well, the university can do this or that, but then no, you don't have time. Mostly, I think about time management and perhaps changing some university policies that allow you to have more time to perform activities other than the academic ones" (FG 3)
		f) "The other thing is also television, social media, sports. I really like football, watching it, not playing it, but watching it a lot and as much as I can. Family and social activities also strengthen you and strengthen the family, which is fundamental." (FG 3)
	COVID-19	*g) "With COVID it increased, for me... at first it was the shock of the teachers not being prepared, anyone, nobody was really prepared, so what did they do? More, more and more load, more work, more work, more work, more work, then it was a bigger burden." (FG 2)*
		h) "In the social aspect, it didn't affect me so much because I don't go out often, so it didn't affect me so much, but in the clinical aspect, yes, for me it is… Now I am already used to it, but this whole business of using all those biosafety things, for me it was exasperating, not to be able to speak well with my patients, they not being able to look at me in the eye, I mean, to suffocate with that, that did affect me. But now I already got used to it (laughter)" (FG 2)
**2) A rollercoaster: the postgraduate program like a personal life and**	Overload	*a) "In my case, I had to stop working for a year and a half, or the first three semesters because the academic load didn't really allow me to fulfill, let's say, my academic duties. It really forced me to that and also to give up family moments, personal moments with my partner" (FG 3)*

**an academic project**	Teacher-student relationship	*b) "Also it would be ideal if teachers recognized postgraduate students as professionals and not only students, I mean, if they could see them maybe not like an equal per se because ultimately it's not quite an equal, but like a professional, which is, a person who went through five years of undergraduate studies, that has a different perception of life, that is not an immature undergraduate student, I mean that vision from teachers… or that treatment from teachers towards residents" (FG 1)*
		c) "I also felt changes in the treatment from the teachers with the students. Maybe in the clinical part teachers are a little stricter and often times learning doesn't mean treating badly, often times you learn easier in other ways, and you start disliking things or you don't do them out of fear but out of responsibility" (FG 2)
	Respect for non-academic spaces	*d) "And the second thing is that each postgraduate program, let's say all the teacher's academic group and on the administrative side, they should keep in mind to respect the non-academic spaces of each student, starting off from the fact that all of us who register into a postgraduate program are adults, who must organize our own time, so they shouldn't expect to take up those spaces that can get to be non-academic. For example, a teacher shouldn't text you in WhatsApp on a Saturday or Sunday night asking for postgraduate-related things, but instead obviously consider that although we have certain responsibilities regarding the postgraduate program, also like respect each space that can be defined as non-academic, such as weekends, Saturday night, Sunday, when you could be doing other things and not be focusing exclusively on the matter of the postgraduate program" (FG 3)*
	Adaptation process	*e) "That question at this moment, truth be told, today, I feel super happy and fulfilled, if that question had been done last semester, I can tell you that my answer would have been completely unhappy, because that's how I felt, unhappy. I was telling my husband to tell me what happiness was to him, and so he told me it was everything that I didn't have: time, time to be with my family, being able to rest, being able to sleep well. But now, as we get to our last year, because we are in our last year of residency, we've had less academic load, less assignments, what the other participant was saying, like rather more clinical practice, that distress of looking for patients, of not knowing if we are going to get them is no longer there, because we are already more balanced, so right now, yes, I feel happy, content." (FG 1)*
	Personal and professional step	*f) "The meaning that the postgraduate program has for me it's like a step, one more step in my life as a professional and as a person, and everything that you learn in this life is a win, no matter what the subject is. Here specifically is about the profession that I chose and so I think that's going to make me better from now on". (FG 3)*
